# Identification of Novel Small Molecule Ligands for JAK2 Pseudokinase Domain

**DOI:** 10.3390/ph16010075

**Published:** 2023-01-04

**Authors:** Anniina T. Virtanen, Teemu Haikarainen, Parthasarathy Sampathkumar, Maaria Palmroth, Sanna Liukkonen, Jianping Liu, Natalia Nekhotiaeva, Stevan R. Hubbard, Olli Silvennoinen

**Affiliations:** 1 Faculty of Medicine and Health Technology, Tampere University, 33014 Tampere, Finland; 2 Institute of Biotechnology, HiLIFE Helsinki Institute of Life Science, University of Helsinki, 00014 Helsinki, Finland; 3 Department of Biochemistry and Molecular Pharmacology, New York University Grossman School of Medicine, New York, NY 10016, USA; 4 Surrozen, Inc., South San Francisco, CA 94080, USA; 5 Single Cell Core (SICOF), Department of Medicine Huddinge, Karolinska Institutet, SE-14157 Huddinge, Sweden; 6 Karolinska High Throughput Center, Department of Biosciences and Nutrition, Karolinska Institutet, SE-14186 Huddinge, Sweden; 7 Science for Life Laboratory, Biochemical and Cellular Assay Facility, Drug Discovery and Development Platform, Department of Biochemistry and Biophysics, Stockholm University, Solna, SE-17121 Stockholm, Sweden; 8 Fimlab Laboratoriot Oy Ltd., 33013 Tampere, Finland

**Keywords:** JAK inhibitor, JAK2 V617F, myeloproliferative neoplasm, cytokine signaling, pseudokinase

## Abstract

Hyperactive mutation V617F in the JAK2 regulatory pseudokinase domain (JH2) is prevalent in patients with myeloproliferative neoplasms. Here, we identified novel small molecules that target JH2 of JAK2 V617F and characterized binding via biochemical and structural approaches. Screening of 107,600 small molecules resulted in identification of 55 binders to the ATP-binding pocket of recombinant JAK2 JH2 V617F protein at a low hit rate of 0.05%, which indicates unique structural characteristics of the JAK2 JH2 ATP-binding pocket. Selected hits and structural analogs were further assessed for binding to JH2 and JH1 (kinase) domains of JAK family members (JAK1-3, TYK2) and for effects on MPN model cell viability. Crystal structures were determined with JAK2 JH2 wild-type and V617F. The JH2-selective binders were identified in diaminotriazole, diaminotriazine, and phenylpyrazolo-pyrimidone chemical entities, but they showed low-affinity, and no inhibition of MPN cells was detected, while compounds binding to both JAK2 JH1 and JH2 domains inhibited MPN cell viability. X-ray crystal structures of protein-ligand complexes indicated generally similar binding modes between the ligands and V617F or wild-type JAK2. Ligands of JAK2 JH2 V617F are applicable as probes in JAK-STAT research, and SAR optimization combined with structural insights may yield higher-affinity inhibitors with biological activity.

## 1. Introduction

JAK2 belongs to the Janus kinase family of cytoplasmic non-receptor tyrosine kinases (JAK1-3, TYK2) and functions as a critical mediator of signaling for hematopoietic cytokines and hormones [1]. JAK2, like other JAK family members, comprises a catalytically active C-terminal tyrosine kinase domain (JH1), a regulatory pseudokinase domain (JH2), and a Src homology-2 (SH2)-like domain that, together with the N-terminal FERM (N-terminal 4.1, ezrin, radixin, moesin) domain, forms the major receptor interaction moiety. Both biochemical and clinical studies have identified the JH2 domain as a critical regulatory domain in JAKs [2,3,4,5,6,7,8]. The important role of the JH2 domain is emphasized by the fact that over 30 different mutations in JAK2 JH2 have been shown to cause, or be linked to, hematological diseases (reviewed in [9]). The most frequent somatic mutation, V617F, results in a constitutively active JAK2 and is found in >95% of polycythemia vera cases and ~50% of essential thrombocythemia and primary myelofibrosis cases [10]. 

Previous studies have demonstrated that JAK2 JH2 binds ATP and possesses regulatory autophosphorylation activity on Ser523 and Tyr570 [3]. Further, the inhibition of ATP binding to JAK2 JH2 via mutagenesis displayed selective inhibition of hyperactive pathogenic JAK2 mutants [11]. These findings suggest that inhibiting ATP binding to JAK2 JH2 by small molecules might inhibit hyperactive JAK2 signaling. The ATP-binding pocket of JH2 has recently been validated as a viable drug target by a TYK2-selective inhibitor, deucravacitinib, which, via targeting of the JH2 ATP-binding pocket (of TYK2 and JAK1), selectively inhibits TYK2-mediated signaling [12]. Deucravacitinib was recently approved by the FDA for the treatment of psoriasis and is currently under clinical evaluation for psoriatic arthritis (Phase III) and systemic lupus erythematosus (Phase II). Most JAK inhibitors target the active, highly conserved JH1 domain with variable JAK and/or cytokine pathway selectivity, are not curative, and result in various side effects. 

The present study aimed to identify small molecule inhibitors that target the ATP-binding site in JH2 of JAK2 V617F. We performed large-scale in vitro screens for detection of small molecular weight (smw) compound binders against recombinant JAK2 JH2 V617F protein. The identified hits and their structural analogs were profiled for JAK and kinase/pseudokinase domain selectivity and assessed for inhibition of JAK2 V617F activity in MPN model cell lines. Finally, the binding of selected hits and hit analogs to JAK2 JH2 wild-type and V617F mutant were characterized with X-ray crystallography.

## 2. Results

### 2.1. Small-Scale Pilot Screen Compared TSA and FP as Screening Methods

Small-scale pilot screens were performed to detect binders for JAK2 JH2 V617F recombinant protein using thermal shift assay (TSA) and/or fluorescence polarization (FP) screening techniques in 384-well plate format (Table 1). A collection of 4200 compounds was screened by TSA at 10 µM concentration and 2100 small molecules by TSA and FP at 20 µM concentration. Libraries consisted of known kinase inhibitors, nucleotide/nucleoside analogs and clinical compounds. TSA measures protein thermal unfolding and ligand binding can be observed as a shift in the unfolding temperature (ΔTm) [13,14]. The TSA screens identified six compounds that induced shift in the unfolding temperature of recombinant JAK2 JH2 V617F at or above 2 °C, four of which were identified from collection screened only by TSA and two, JNJ-7706621 (**1**) and AT9283 (**2**), from collections screened by TSA and FP. Compounds **1** and **2** are known kinase inhibitors and their binding to JAK2 JH2 has been reported previously [15,16,17]. Fluorescence polarization assay, which detects ligands that out-compete a fluorescent tracer, Bodipy FL-ATP, from the ATP-binding site, was then applied as an alternative screening method for 2100 compounds included also in TSA-screen. Small-scale FP screen resulted in identification of the TSA-screen hits **1** and **2** and additional 28 hits (Table 1). The hit rate in FP screen (1.5%) was significantly higher than in TSA screen (0.096%) and thus FP as a more sensitive method was selected for large-scale screening.

### 2.2. Summary of the Screens

**Figure 1:**
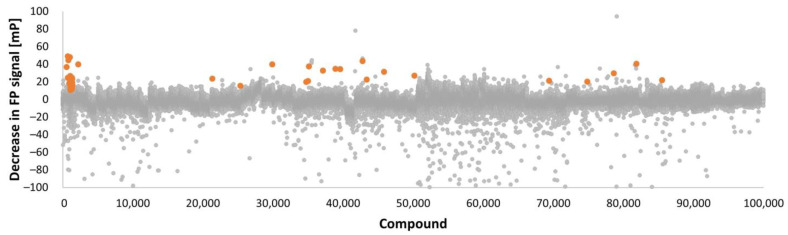
Fluorescence polarization signal in screening. Data presented is decrease in FP [mP] for each 103,400 compounds screened. Verified 51 hits are marked with orange color.

After the pilot screens in 384-format, a follow-up screen of 101,300 smw compounds was performed at Karolinska High-Throughput Center using FP in 1536-format (Table 1). A total of 70 plates containing compounds from targeted and diverse libraries were screened by FP. Median Z-factor was 0.62 (range of 0.21–0.78; average 0.60), which indicate general robustness of the screening. Signal decrease in FP screens, indicating a displacement of the tracer from the ATP pocket, is presented as a scatter blot in Figure 1 with verified hits marked as orange dots. Fifty-one hits were verified in dose–response as binders of JAK2 JH2 V617F from FP screens, and an additional four hits were verified from TSA pilot screen (Table 1). 

Majority of the hits were from Otava nucleotide/nucleoside analogs compound set (22 hits), followed by diverse compound set from Maybridge (9 hits) and kinase targeted libraries from Biomol (5 hits) and Uni Copenhagen (4 hits) (Table 1). Few binders of JAK2 JH2 V617F were found from diverse compound sets from Enamine (3 hits), Specs (3 hits) and ChemBridge (2 hits), known-drugs collection from Prestwick (3 hits), clinical bioactives collection from National Institutes of Health (NIH; 2 hits), kinase-targeted library from SelleckChem (1 hit), and fragment set from Org Pharm Chem (1 hit). Hit rate in the screening was low, 0.051%, which indicates low affinity of the small molecules in general for the JAK2 pseudokinase domain and the unique nature of JAK2 JH2 ATP pocket, further suggesting that the ATP binding pocket of JAK2 JH2 may be targeted with high specificity.

### 2.3. Primary Hit Follow-Up

**Figure 2:**
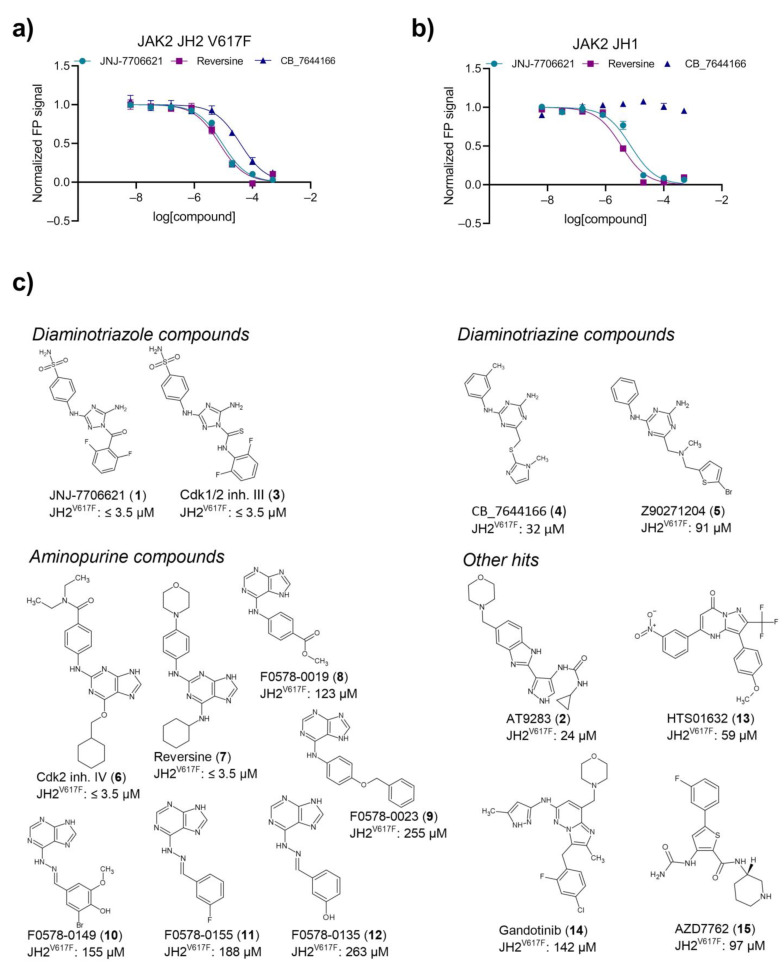
Dose-response data and chemical structures of selected hits. (**a**) Normalized dose–response data JNJ-7706621 (**1**), reversine (**7**), CB_7644166 (**4**) from fluorescence polarization assay for recombinant JAK2 JH2 V617F. Binding of compounds in ATP pocket of proteins is observed as signal decrease. (**b**) Normalized dose–response data JNJ-7706621 (**1**), reversine (**7**), CB_7644166 (**4**) from fluorescence polarization assay for recombinant JAK2 JH1. (**c**) Chemical structure and IC50 value for binding against JAK2 JH2 V617F of confirmed hits. Hits were grouped based on chemical structure as diaminotriazole compounds, diaminotriazine compounds, aminopurine compounds, and other hits.

A set of 15 commercially available synthetic hit compounds (**1**–**15**) were selected for dose–response binding analyses against recombinant JAK2 JH2 V617F, JAK2 JH2 wt, and JAK2 JH1 proteins. Dose–response FP data for JAK2 JH2 V617F protein (Figure 2a) indicate higher-affinity binding of compound **1** and reversine (**7**) than CB_7644166 (**4**). Compounds **1** and **7** targeted also the ATP-binding site of JAK2 JH1 domain, whereas no binding of **4** to the JH1 domain was observed (Figure 2b). Hits were categorized into four groups according to their chemical structures, i.e., diaminotriazole compounds, di-amino triazine compounds, aminopurine compounds, and other hits (Figure 2c). Four hits, i.e., diaminotriazole compounds **1** and Cdk1/2 inhibitor III (**3**), and aminopurine analogs Cdk2 inhibitor IV (**6**) and **7** bound JAK2 JH2 (V617F and wt) and JH1 domains with IC50 values at or below lower detection limit of assay (3.5 µM) (Figure 2c; Supplementary Table S1). Diaminotriazine compounds **4** and Z90271204 (**5**) and phenylpyrazolo-pyrimidone compound HTS01632 (**13**) possessed JH2-selective binding over JH1 with IC50 from 32 to 91 µM for JH2 V617F (Figure 2c). 

### 2.4. JH2 and JH1 Domain Targeting of Selected Hit Analogs

Compounds analogous to identified hits were searched from PubChem and ZINC databases [18,19]. Identified 15 diaminotriazole analogs (Supplementary Table S2), 24 diaminotriazine analogs (Supplementary Table S3), 9 phenylpyrazolo-pyrimidone analogs (Supplementary Table S4), and 9 pyrazolyl-formamide analogs (Supplementary Table S5) were assessed for binding to recombinant JAK2 JH2, JH2 V617F and JH1, and JAK1 JH2 proteins. 

**Figure 3:**
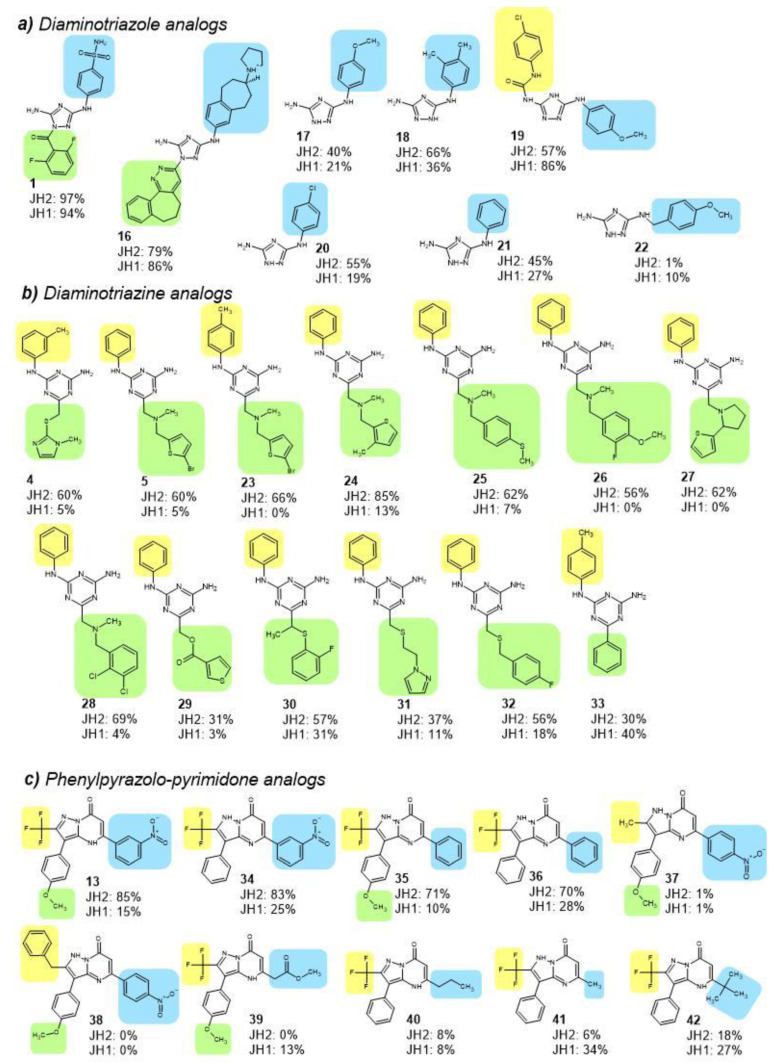
Chemical structures of selected analogs and binding to JAK2 JH2 and JH1 domains. (**a**) Diaminotriazole analogs, (**b**) diaminotriazine analogs, and (**c**) phenylpyrazolo-pyrimidone analogs. Subgroup R1 marked with yellow, R2 with green and R3 with blue background. Binding data are inhibition-% of tracer binding to recombinant JAK2 JH2 and JH1 domains at 100 µM analog concentration.

Diaminotriazole compounds **16**–**21,** in addition to primary hit **1**, demonstrated substantial binding to JAK2 JH2 domain ATP-pocket, i.e., competition of fluorescent tracer was at or above 30% at 100 µM compound concentration (Figure 3a). The initial hit **1** among the diaminotriazine compounds bound tightest to both JH2 and JH1 domains, followed by analogs **16**, **18** and **19**, which resulted in 79%, 66% and 57%, respectively, inhibition of tracer binding to JH2 domain at 100 µM concentration. Compounds **16** and **19** engaged equally JH2 and JH1 domains, whereas analogs **17**, **18**, **20** and **21** possessed JH2-selective binding (2- to 3-fold). JH2-selective diamino triazole analogs shared a similar chemical structure with hydrogen as subgroups R1 (yellow background in Figure 3a) and R2 (green background), and phenyl group with substituents at m- and/or p-positions as R3 (blue background). Difference in the binding characteristics of analogs **17** and **22** indicated that an extra carbon prior to phenyl group at R3 position of **22** is not beneficial for binding to JH2 or JH1 domains (Figure 3a). 

Diaminotriazine compounds **23**–**33** were found to bind JAK2 JH2 domain (Figure 3b) generally with selectivity over JH1, except for analog **33**, which bound JH1 domain more effectively than JH2. **33** has a smaller R2 subgroup, phenyl ring, than the JH2-selective triazine analogs. Level of binding to JH2 domain by analogs **23**–**28** was equal or stronger compared to initial hits **4** and **5**. In general, the binding of diaminotriazine analogs was stronger to JH2 domain of JAK1 compared to JH2 domain of JAK2 (Supplementary Table S4). 

Phenylpyrazolo-pyrimidone compounds **34**, **35**, and **36**, analogs of hit **13**, engaged JAK2 JH2 domain with selectivity over JH1 (Figure 3c). Analog **41** preferred JH1 in binding and analogs **37**–**40** and **42** bound neither JH2 nor JH1 domain (inhibition of tracer binding below 30% at 100 µM). The compounds that targeted JH2 domain shared a trifluoro-group as R1 (Figure 3c). Although presence or absence of nitro-substituent at m- or p-position of phenyl ring as R3 or methoxy-group as R2 did not seem to have a major effect on binding to JH2 or JH1 domains, the aromatic nature of R3 subgroup was beneficial for binding to JAK2 JH2.

Nine analogs of hit **2,** pyrazolyl formamide compounds, were identified. Two analogs were observed to bind JAK2 JH2 domain, although the compounds targeted JH1 domain with higher potency and binding level to JH2 was lower compared to initial hit **2** (Supplementary Table S5). JH2-targeting of pyrazolyl-formamide analogs was equal or tighter for JAK1 compared to JAK2.

### 2.5. JAK Selectivity and Inhibition of MPN Cell Lines of Selected Binders

Fluorescence polarization assay with enhanced, nanomolar-scale sensitivity by applying a tighter-binding tracer, Bodipy FL-labeled JNJ-7706621 [20] was performed to study JAK and domain selectivity of selected hits and hit analogs. Selection was based on strength of binding to JAK2 JH2 and/or JH2-selectivity over JH1, and the assessment included diaminotriazole compounds **1** and **18**, diaminotriazine compounds **4** and **24**, aminopurine compound **7**, phenylpyrazolo-pyrimidone analogs **34** and **36**, and pyrazolyl-formamide compound **2**. Inhibition of MPN model cell viability was also measured. 

The initial hits **1** and **2** possessed highly JH1-selective binding over JH2 on JAK2, whereas JH1-selectivity of **7** was minor (Table 2). These compounds targeted JH1 and JH2 domains of all JAK family members. Interestingly compound **7** bound JAK1 and TYK2 JH2 domains tighter than JAK2 or JAK3 JH2 domains, and binding to JAK1 and TYK2, unlike to JAK2 or JAK3, was slightly (2-fold) JH2-selective over JH1. Compounds **1**, **2**, and **7** also inhibited viability of MPN model cell lines HEL and SET-2 (Table 2), both of which express JAK2 V617F (homozygous in HEL, heterozygous in SET-2), and murine pro-B cells Ba/F3 carrying either hJAK2 wildtype or V617F (Supplementary Table S6) at submicromolar-micromolar range. 

Diaminotriazole analog **18**, diaminotriazine compounds **4** and **24**, and phenylpyrazolo-pyrimidone analogs **34** and **36** targeted JAK2 JH2 with selectivity over JH1, although the affinity of these compounds to JH2 domain was micromolar and thus lower compared to the JH1-targeting compounds **1** and **2**. Interestingly diaminotriazine compounds (**4** and **24**) were observed to target JAK2 JH2 V617F 2- to 3-fold stronger than JAK2 JH2 wt. The JH2-selective compounds additionally targeted one to three other JAK JH2s with IC50 below 100 µM but did not inhibit viability of MPN cell lines (upper detection limit IC50 = 40 µM).

### 2.6. Protein-Ligand Crystallization

**Figure 4:**
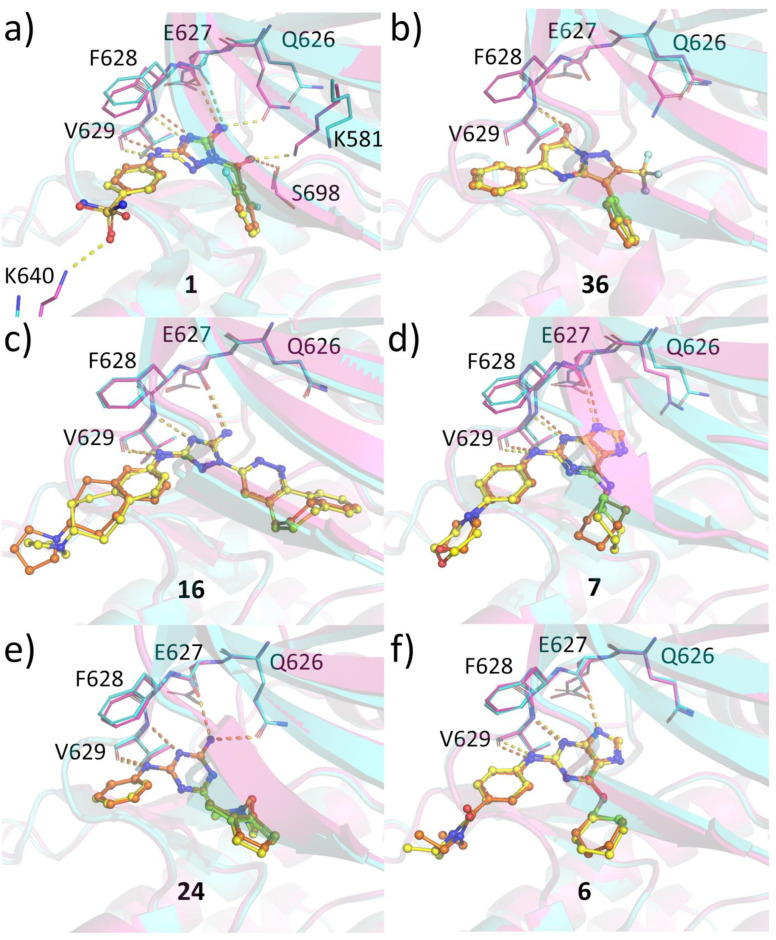
Crystal structures of selected hits and hits analogs with JAK2 JH2 wild-type and V617F mutant. The residues participating in hydrogen bonding and the gatekeeper Gln626 are shown as sticks. The ligand complexes of WT and V617F are superposed together. (**a**) Compound **1** (RMSD for aligned Cα atoms: 0.43). (**b**) Compound **36** (RMSD for aligned Cα atoms: 0.133). (**c**) Compound **16** (RMSD for aligned Cα atoms: 0.371). (**d**) Compound **7** (RMSD for aligned Cα atoms: 0.378). (**e**) Compound **24** (RMSD for aligned Cα atoms: 0.243). (**f**) Compound **6** (RMSD for aligned Cα atoms: 0.43). For JAK2 JH2 WT, the structure is shown in turquoise, the ligand and hydrogen bonds in orange. For JAK2 JH2 V617F, the structure is shown in magenta, the ligand and hydrogen bonds in yellow. JAK2 JH2—**1** complex structure has been determined previously (pdb code 5WIN).

To gain insights on the compound binding modes to JAK2 JH2 and to analyze whether any differences could be observed between binding to WT and V617F, we determined the crystal structures of selected small molecules with JAK2 JH2 WT and V617F. All compounds anchor to the hinge region and extend towards the Gln626 gatekeeper and towards the G-loop (Figure 4). All compounds bind to the hinge via three hydrogen bonds (H-bonds) with the exception of **36**, which makes only one hydrogen bond to the backbone amide nitrogen of Val629. The rest make two additional H-bonds to backbone carbonyl oxygens of Val629 and Glu627. In addition, **24** directly hydrogen bonds to the side chain of the Gln626 mimicking the interaction made by adenine base in ATP bound structure (pdb code 4FVR) [21]. Additionally, **1** makes this same interaction but only in the V617F structure. In addition, **1** forms an H-bond with the side chain of Lys640 from the αD helix. This interaction is also not present in the WT structure. **1** interacts with the side chain of Ser698 in the WT structure but in V617F structure this serine assumes two conformations and instead of interacting with the serine, **1** forms a hydrogen bond with Lys581, which is shifted closer to the compound. Overall, the binding modes are highly similar between WT and V617F structures and no major differences can be seen that would explain the differences in the observed IC50 values. Differences observed in the complexes with **1** stem from the conformation of the Gln626, the αD lysine and differences at the back of the binding pocket. It should be noted that the conformation of the gatekeeper displays rather large variations in the structures, and it is unclear what the contribution of this interaction to the binding affinity is, especially since diaminotriazole analog **16**, which also contains amide nitrogen at this position (actually even closer to the Gln626) does not make the gatekeeper H-bond. The αD lysine, in turn, is solvent exposed and rather distal from the ATP pocket, and it is unlikely that this interaction significantly contributes to the binding affinity. The variation in the hydrogen bonding pattern in the back of the pocket (Ser698 in WT vs. Lys581 in V617F) might reflect differences in the dynamics of the pocket between the structures but altogether the differences in hydrogen bonding with **1** is not reflected on IC50 values. For two compounds, **4** and **23**, no structure with JAK2 JH2 WT was obtained. They both make the three hinge hydrogen bonds, and also interact with the Gln626 gatekeeper (Supplementary Figure S1).

To find an explanation for the JH2 over JH1 selectivity for some of the compounds, we compared the structures of JAK2 JH1 with the complexes of **36**, **23**, and **4** (Supplementary Figure S2). Overall, the ATP-pockets of JH2 and JH1 are well-conserved. However, certain regions can explain the observed differences in IC50 values. First, the methionine gatekeeper of JH1 does not offer possibilities for hydrogen bonding unlike the polar glutamine. Second, the β3 Leu579, which flanks the ATP adenine mimicking moiety in the compounds, is replaced by alanine in JH1. Leucine provides a hydrophobic environment for this region possibly influencing the binding affinity. Third, the compounds also extend towards the glycine-rich G-loop, which in the JH1 structure would clash with the compounds. However, G-loop is often highly mobile in kinases, and it is possible that the G-loop region of JH1 could accommodate these compounds without a negative effect in the binding affinity.

## 3. Discussion

JAK kinases share a tandem kinase domain structure consisting of an active tyrosine kinase domain and a regulatory pseudokinase domain that binds ATP but is generally devoid catalytic activity [2,3,4,5]. JAK2 JH2 exceptionally possesses non-canonical phosphotransfer activity via phosphorylation of two negative regulatory sites, Ser523 and Tyr570 to maintain low activity in the absence of cytokine stimulation [3,6]. The JH2 domain has a key role in mediating both negative and positive regulation of JAK kinase activity and numerous disease mutations locate in JH2, including the prevalent hyperactivating JAK2 V617F in MPN. V617F induces cytokine-independent receptor dimerization, which leads to cytokine-independent activated JAK2-STAT5-signaling [22].

The pseudokinase domain of JAKs may theoretically be targeted with higher specificity compared to the highly conserved kinase domain. Furthermore, the current JAK2-inhibitors in treatment of MPNs do not induce remission and thereby alternative drug approaches are needed. The pseudokinase domain of JAKs has raised interest in recent years. Series of TYK2 pseudokinase binders that selectively suppress TYK2-mediated signaling has been developed by Bristol Myers Squibb [12,23], which recently led to FDA-approval of deucravacitinib for the treatment of plaque psoriasis. The JAK1 pseudokinase domain has also been targeted by small molecules [24]. Further, targeting of JAK2-mediated signaling via JH2 has been previously approached via screening of up to 1000 small molecules for binding against JH2 domain [15,25] and via virtual screening approach [26]. Here, we describe novel JH2-binding chemical structures obtained from extensive screening of nearly 110,000 compounds. Unsurprisingly, almost half of the identified JH2-domain ATP-pocket binders, 22 out of 55, were nucleotide analogs, whereas only 10 hits were identified from kinase-targeted compound collections of 5,600 small molecules, which indicates the unique nature of ATP-pocket of JAK2 pseudokinase domain in the kinome. Seventeen binders were from diverse compound sets.

JNJ-7706621 (**1**) and AT-9283 (**2**) were also observed as JAK2 JH2 binders in a previous screening by Puleo and coworkers [15]. Interestingly, a recently published virtual screening for binders to JAK2 JH2 led to identification and development of a JH2-selective aminoanilinyltriazine compound series by Jorgensen and Schlessinger groups [26]. Our diaminotriazine hits share the aminoanilinyltriazine core structure and were confirmed to have JH2-selectivity over JH1. Data in the present study and that by Jorgensen group indicate that aminoanilinyltriazine compounds might possess slightly (2- to 3-fold) stronger binding to V617F than wild-type. Unfortunately, we were able to obtain a good-quality crystal structure of diaminotriazine compound **23** in complex only with JAK2 JH2 V617F and thus basis for the observed minor, but consistent difference remains elusive. 

Phenylpyrazolo-pyrimidone compounds identified in the present study are a novel finding for JH2-selective binders of JAK2. The compounds have previously observed to function as potassium channel (Kv7/KCNQ) activators [27]. The affinity of phenylpyrazolo-pyrimidone compounds as well as of other JH2-selective small molecules to JAK2 JH2 was rather low, in the micromolar range. Considering that generally 10-200-fold amount of inhibitor is needed for cellular effects, it is not surprising that no inhibitory effects with the low-affinity JH2-selective binders were observed in MPN cells, where detection of inhibitory effects is limited due to compound solubility issues to IC50 ≤ 40 µM. Although mutagenesis studies [11] suggested that inhibition of ATP binding to JAK2 JH2 by small molecules might inhibit hyperactive JAK2, the prediction of the cellular effects of JH2-selective small molecules is challenging given the complex regulatory role of the JH2 domain, with both positive and negative effects on JAK activation [2,3,4,5,6,7,8,28]. SAR optimization is needed to enhance affinity of the novel JH2-selective hit structures in order to evaluate their cellular effects. The compounds could further be useful as JH2-selective probes in JAK research. 

**Figure 5:**
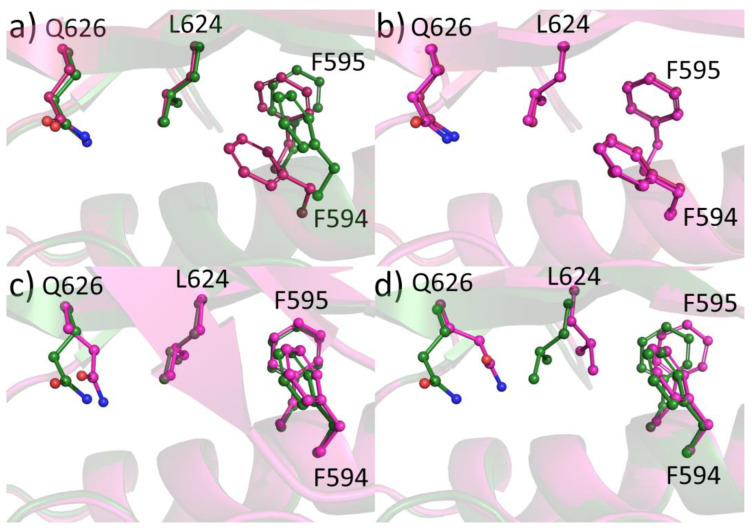
Differences induced by compound binding to the conformation of Phe594 and Phe595. (**a**) Comparison of JAK2 JH2 WT (PDB code 4FVQ, green) and JAK2 JH2 V617F (PDB code 4FVR, pink). (**b**) Comparison of JAK2 JH2 V617F-**24** (magenta) and JAK2 JH2 V617F (pink). (**c**) Comparison of JAK2 JH2 V617F-**6** (magenta) and JAK2 JH2 WT (green). (**d**) Comparison of JAK2 JH2 V617F-**16** (magenta) and JAK2 JH2 WT (green).

Structural analysis of compound binding modes to JAK2 JH2 WT and V617F mutant revealed no major differences in the binding modes with the exception of compound **1**. However, these differences did not translate into potency differences in the binding assays. Since the compound binding does not induce significant changes in the ATP pocket, we compared the regions around residue 617 and the helix Cα between WT and V617F structures. It has been noted before [25] that compound binding to JAK2 JH2 V617F can disrupt the aromatic packing of F617-F595-F594 and the conformation of F595 and F594 can be reverted back to the JAK2 JH2 WT conformation (Figure 5a,b). We compared our eight V617F complexes with JAK2 JH2 WT (PDB code 4FVQ) and V617F (PDB code 4FVR) and noticed that in most structures the V617F-like pi-stacking of Phe595-Phe594 was intact. In structures complexed with **6**, **36**, and **16**, a WT-like conformation for the phenylalanines was observed. In the complexes with **36** and **16**, the switch towards the WT-like conformation seems to start from the shifting of Gln626 gatekeeper position, which in turn affects the conformation of Leu624. This leucine forces both Phe594 and Phe595 to a new conformation (Figure 5c,d). However, the Gln626-Leu624 switch is not present in the complex with **6** but the structure still assumes a WT-like Phe594-Phe595 conformation. Interestingly, the packing of F617 against F595 observed in the V617F-ATP complex was not evident in any of our structures and the residue often displayed poor electron density suggesting high flexibility. It appears to be possible to affect the conformation of the V617F region with small molecules, especially ones disturbing the position of the gatekeeper. However, before a possible biological effect of these compounds can be evaluated, a higher selectivity over the JH1 domain is required. Recently developed JAK2 JH2 targeting diaminotriazoles [29] display very high selectivity over JH1 but did not inhibit cellular STAT5 phosphorylation at concentrations lower than required for JH1 inhibition. Compounds from this series whose structure was determined (with JAK2 JH2 WT domain) did not affect the Gln626-Leu624 axis and therefore unlikely induce WT-like conformation in the V617F mutant structure. Combining high selectivity with the ability to reverse the F594-F595 back to WT-like conformation could be a potential approach to modulate pathogenic JAK2 signaling with JH2 specific compounds.

## 4. Materials and Methods

### 4.1. Cloning, Production and Purification of Proteins Used in This Study

Human JAK2 JH2 (503-827), JAK2 JH1 (836-1132) D976N, JAK1 JH2 (553-836), JAK1 JH1 (866-1154) D1003N, TYK2 JH1 (886-1187) D1023N, JAK3 JH1 (811-1124) D949N, and JAK2 JH2 (536-812) V617F-W659A-W777A-F794H were cloned into pFASTBAC1 vector (Invitrogen) with a C-terminal hexa histidine-tag. Mutations were introduced by site-directed mutagenesis using QuikChange-protocol with specifically designed primers and verified by using Sanger sequencing. Preparation of other JAK constructs has been reported previously [2,5,21].

Recombinant human JAK2 JH2 V617F, JAK2 JH2 (536-812) W659A-W777A-F794H, JAK2 JH2 (536-812) V617F-W659A-W777A-F794H, JAK2 JH2 (536-812) V617F-W777A-F794H, JAK2 JH2, JAK2 JH1 D976N, JAK1 JH2, JAK1 JH1 D1003N, JAK3 JH2 (511-790), JAK3 JH1 D949N, TYK2 JH2 (564-876), and TYK2 JH1 D1023N proteins with C-terminal histidine tag were expressed in High Five^TM^ or Sf9 insect cells. Proteins were purified using Ni-NTA-affinity and size exclusion chromatography (SEC) as described earlier [30]. JAK2 JH2 V617F protein used in screening approaches was Ni-NTA-affinity purified, whereas the recombinant proteins for further assessments were affinity- and SEC-purified.

### 4.2. Screening

Small-scale pilot screen of 2100 smw compounds from kinase-targeted (Biomol and SellechChem), nucleotide/nucleoside analogs (Otava) and clinical bioactives (NIH) collections (Table 1) for binders of recombinant JAK2 JH2 V617F protein was performed in 384-well plate format using thermal shift assay (TSA) and fluorescence polarization (FP) technologies at Karolinska High-Throughput Screening Center. Additionally, a small-scale screen of 4200 small molecules was performed by TSA at Novo Nordisk Foundation Center for Protein Research, University of Copenhagen. Thermal shift assay measures protein thermal stability and is applicable for identification of binders that stabilize (or de-stabilize) the protein. For the assay, reaction components 6x Sypro Orange, 2 µM recombinant JAK2 JH2 V617F, 1 mM MgCl2, 20 mM Tris-Cl pH 8.5, 500 mM NaCl, and 20% glycerol were mixed and dispensed by Beckmann Biomek FX^p^ automation workstation in volume of 5 µL per well, on top of which smw compounds (final concentration 10-20 µM), DMSO (negative control) or VI16832 (positive control) were dispensed by Echo555 acoustic dispenser (Beckman Coulter). TSA run with temperature increase of 1 °C per 30 s with signal detection after each increase was performed using Roche LightCycler480. Melt temperatures (Tm) were calculated and compounds that resulted in Tm increase at or above 2 °C were identified as hits. To compare two screening methods, the pilot screen of 2100 compounds was also performed using FP that was based on a fluorescent tracer Bodipy FL-ATP (Thermo Fisher Scientific), which binds to the ATP-pocket of JAK2 JH2 V617F. ATP-competitive binding of a smw compound resulted in FP signal decrease. Library compounds, DMSO or JNJ-7706621 (positive control; a hit from TSA pilot screen) were dispensed on empty screening plates by Echo555 acoustic dispenser (Beckman Coulter), followed by dispensing the reaction mixture containing 7 µM protein and 40 nM Bodipy FL-ATP in sample buffer (10 mM MgCl_2_, 20 mM Tris-Cl pH 8.5, 150 mM NaCl, 0.01% Brij-35, 20% glycerol) by Multidrop dispenser (Thermo Scientific). FP was detected by measuring fluorescence (ex. 480/30 nm, em. 535/40 nm S-pol and P-pol) in EnVision multilabel plate reader (PerkinElmer) equipped with a FITC FP package. 

Large-scale screens were performed in 1536-well plate format by applying FP as a screening method. Reaction mixture composed of 8 µM protein, while other components were the same as in pilot screen described above. Reaction volume was 2 µL and binding of smw-compounds was assessed at 25 µM compound concentration. The sample plates were stored at +4 °C up to 30 min, incubated at RT for 5-10 min, and FP was detected as in pilot screen described above. Altogether 103,400 smw compounds were screened by FP from multiple libraries including kinase-targeted compounds, nucleotide/nucleoside analogs, known drugs, small molecule fragments, clinical bioactive compounds, natural derivatives, and other diverse compound sets (Table 1).

Z-factor was calculated for each screening plate according to Equation (1):

(1) $$\;Z^{\prime }=1-3*\frac{\mathrm{stdev}\left(\mathrm{neg}.\;\mathrm{ctrl}\right)+\mathrm{stdev}\left(\mathrm{pos}.\;\mathrm{ctrl}\right)}{\mathrm{average}\;\left(\mathrm{neg}.\;\mathrm{ctrl}\right)-\mathrm{average}\left(\mathrm{pos}.\mathrm{ctrl}\right)}$$

where stdev and average are standard deviation and average of FP signal. Decrease in FP signal was calculated for each compound by subtraction of signal in compound well from average signal in negative control wells. Calling of preliminary hits was performed for each plate, and compounds that resulted in a decrease in FP signal greater than 6 times the standard deviation in the neg. ctrl wells were identified as preliminary hits. The preliminary hits were verified at concentrations 12.5 µM, 25 µM and 50 µM in FP assay at the abovementioned assay conditions. Preliminary hits that resulted in concentration-dependent decrease in FP were verified as hits. 

### 4.3. Compounds

Selected verified hits were ordered for further testing. Supplier details for hits and product details for hit analogs are listed in Tables S7 and S8, respectively. All smw compounds in the study were synthetic.

### 4.4. FP Binding Assay

Binding of hits and their structural analogs was first characterized in five-fold dilutions at concentration range of 6 nM to 500 µM for JAK2 JH2 V617F, JAK2 JH2 wild-type and JAK2 JH1 D976N in 384-well plate format and assay parameters as in pilot screen. Fluorescence polarization values obtained were fitted against log[inhibitor] in GraphPad Prism to yield IC50 values. Due to micromolar protein-tracer binding affinity (7 µM for JAK2 JH2 V617F, 9 µM for JAK2 JH2 WT, 5 µM for JAK2 JH1) in the assay conditions and thereby micromolar (7 µM) protein amount applied, the assay was sensitive to detect µM IC50 values.

Selected hits or hit analogs were further assessed in a more sensitive FP binding assay that applied Bodipy FL-labeled **1** as a tracer as reported earlier [5]. Concentrations of recombinant proteins were chosen based on protein titration to yield 90% of max signal: 4 nM JAK1 JH1 D1003N, 1.3 nM JAK1 JH2, 5.8 nM JAK2 JH1 D976N, 52 nM JAK2 JH2, 1.1 nM JAK3 JH1 D949N, 1000 nM JAK3 JH2, 5.9 nM TYK2 JH1 D1023N, or 24 nM TYK2 JH2. Compounds were titrated at concentration range of 0.01 to 100,000 nM, and fluorescence polarization values obtained were normalized and pooled from 3-6 individual experiments. Pooled data was fitted against log[inhibitor] in GraphPad Prism to yield IC50 values. IC50 was noted is R^2^ of fit was ≥0.7.

### 4.5. Cell Lines and Cell Viability Assay

Human erythroleukemia (HEL) cells (ATCC; TIB-180) [31] were cultured in RPMI-1640 supplemented with 10% FBS, 1% pen-strep, 1% glutamine, and 10 ng/mL hIL-3, and human megakaryoblastic (SET-2) cells (DSMZ; ACC 608) [32] in RPMI-1640 supplemented with 20% FBS, 1% pen-strep, 1% glutamine, and 10 ng/mL hIL-3. Ba/F3-hJAK2 wildtype and Ba/F3 hJAK2 V617F cells, in which mJAK2 had been replaced with hJAK2 or hJAK2 V617F by CRISPR technology, were a kind gift from prof. R. Skoda (University of Basel). The Ba/F3 cells were cultured in RPMI-1640 medium supplemented with 10% FBS, 1% pen-strep, 1% glutamine and 10 ng/mL mIL-3.

Cells were seeded in white, sterile 384-well plates at 6250 cells/25 µL/well with selected hits or hit analogs at concentration range 0.2 nM–20 µM and incubated for 48 h at 37 °C and 5% CO_2_. Invitrogen alamarBlue cell viability assay (Thermo Fisher Scientific) was performed according to manufacturer’s instructions, and fluorescence (ex. 560 nm, em. 590 nm) was detected by EnVision plate reader. IC50 values were determined by fitting fluorescence signal against log[inhibitor] in GraphPad Prism. Reactions were performed in triplicate and data presented is average of two to three individual experiments.

### 4.6. Protein Crystallography

JAK2 JH2 WT and V617F were crystallized as described previously [21]. JAK2 JH2—small molecule complexes were acquired either by co-crystallization or compound soaking. Co-crystallization was achieved by mixing the small molecule (300 µM) with protein prior to setting up the crystallization drops. To achieve JAK2 JH2—small molecule complexes by soaking, the compounds were soaked at 100 µM concentration into preformed crystals for 24 h in well solution. Before data collection, the crystals were briefly soaked in a well solution supplemented with 20% glycerol and flash frozen in liquid nitrogen. Data were collected at the Advanced Photon Source, Argonne National Laboratory (Illinois, US), on beam line 19-BM. Diffraction data were processed and scaled with the XDS package [33], HKL2000 [34], Xia2 [35], and Aimless [36]. The structures were solved using molecular replacement with JAK2 JH2 V617F mutant (pdb 4FVR) as the starting model. REFMAC5 [37] and phenix.refine [38] were used for refinement and COOT [39] for manual model building. Data collection and refinement statistics are shown in the Supplementary Table S9. JAK2 JH2 V617F-**36** data suffered from translational pseudosymmetry but electron density maps for the protein and the ligand were clear. In JAK2 JH2 V617F-**16** complex, the ligand was not modelled to the B chain due to poor electron density. Coordinates and structure factors have been deposited into the protein data bank (http://www.rcsb.org) with the following accession codes: **8EX1** (JAK2 JH2-**7**), **8BAK** (JAK2 JH2 V617F-**7**), **8EX0** (JAK2 JH2-**6**), **8B8N** (JAK2 JH2 V617F-**6**), **8EX2** (JAK2 JH2-**36**), **8B8U** (JAK2 JH2 V617F-**36**), **8B9H** (JAK2 JH2-**24**), **8B9E** (JAK2 JH2 V617F-**24**), **8BA3** (JAK2 JH2-**16**), **8BA4** (JAK2 JH2 V617F-**16**), **8B99** (JAK2 JH2 V617F-**1**), **8BAB** (JAK2 JH2 V617F-**4**), **8BA2** (JAK2 JH2 V617F-**23**).

## 5. Conclusions

We screened nearly 110,000 small molecules for binding against JAK2 JH2 V617F recombinant protein, which resulted in identification of 55 hits. Low hit rate, 0.05%, indicates distinctive structural features of JAK2 JH2 ATP pocket. Secondary analyses of selected hits and hit analogs resulted with identification of JAK2 kinase/pseudokinase binders and novel low-affinity JH2-selective binders from diaminotriazole, diaminotriazine, and phenylpyrazolo-pyrimidone chemical entities. Structural analysis revealed no major differences in the binding modes of the compounds between JAK2 WT and V617F. Nevertheless, the structures suggest a potential way to selectively inhibit pathogenic JAK2 activity by affecting the position of the gatekeeper residue, which can further induce conformational changes to the mutated F617. SAR optimization could be pursued in future to enhance the affinity of the JH2-selective hit structures in order to gain insights into their putative inhibitory potential. 

## Figures and Tables

**Table 1:** Summary of libraries screened and number of hits. A total number of 107,600 small molecules were screened against recombinant JAK2 JH2 V617F by thermal shift assay (TSA) and/or fluorescence polarization (FP) in 384- or 1536-well plate formats, which resulted in identification of 55 hits.

Library Type	Provider	No. of Compounds	Screening Method	Screening Format	No. of Hits
Kinase-targeted	Uni Copenhagen Biomol SelleckChem Vitas-M	4200 160 190 1000	TSA TSA, FP TSA, FP FP	384- 384- 384- 1536-	4 5 1 0
Nucleotide/ nucleoside analogs	Otava	960	TSA, FP	384-	22
Clinical bioactives	NIH	740	TSA, FP	384-	2
Known drugs	Prestwick	1200	FP	1536-	3
Fragments	Org Pharm Chem	500	FP	1536-	1
Natural derivatives	Timtec Analyticon	3000 1000	FP FP	1536- 1536-	0 0
Diverse	Enamine MayBridge Elite Synergy Specs ChemBridge	28,200 14,400 2300 1900 30,300 17,500	FP FP FP FP FP FP	1536- 1536- 1536- 1536- 1536- 1536-	3 9 0 0 3 2
	SUM:	107,600			55

**Table 2:** JAK- and domain-selectivity of selected hits or analogs and effects on MPN cells. Binding of selected hits or analogs to JAK2 JH2 V617F mutant, JH2 and JH1 domains of JAK family members, and effect on cell viability of MPN cell lines HEL and SET-2. Data is presented as IC50 [µM] obtained by fitting dose vs. normalized response across multiple replica data sets (*n* = 3–9).

Compound		Binding IC50 [µM]									Cell Viability IC50 [µM]	
		JAK2			JAK1		JAK3		TYK2		HEL	SET-2
		JH2 V617F	JH2	JH1	JH2	JH1	JH2	JH1	JH2	JH1		
Diaminotriazole	**1**	0.32	0.31	0.04	0.004	0.08	7.2	0.15	0.14	0.04	5.6	5.8
	**18**	69	67	ND	41	ND	15	ND	ND	ND	ND	ND
Diaminotriazine	**4**	26	68	ND	4.5	ND	ND	ND	27	ND	ND	ND
	**24**	24	70	ND	ND	ND	ND	ND	ND	ND	ND	ND
Aminopurine	**7**	1.9	1.3	0.68	0.15	0.35	4.7	0.30	0.06	0.12	9.3	4.0
Phenylpyrazolo-pyrimidone	**34**	39	14	ND	ND	ND	28	70	9.7	50	ND	ND
	**36**	47	25	ND	ND	ND	ND	ND	32	ND	ND	ND
Pyrazolyl-formamide	**2**	7.7	5.8	0.05	0.11	0.06	11	0.07	0.04	0.02	1.3	0.54

ND, inhibition not detected or IC50 is above upper detection limit (100 µM in binding assay, 40 µM in cell viability assay). Compound abbreviations: **1**, JNJ-7706621; **18**, JS-014C; **4**, CB_7644166; **24**, Z53013220; **7**, Reversine; **34**, HTS02998; **36**, HTS02993; **2**, AT-9283.

## Data Availability

X-ray data is available at Protein Data Bank, Accession codes **8EX1** (JAK2 JH2-**7**), **8BAK** (JAK2 JH2 V617F-**7**), **8EX0** (JAK2 JH2-**6**), **8B8N** (JAK2 JH2 V617F-**6**), **8EX2** (JAK2 JH2-**36**), **8B8U** (JAK2 JH2 V617F-**36**), **8B9H** (JAK2 JH2-**24**), **8B9E** (JAK2 JH2 V617F-**24**), **8BA3** (JAK2 JH2-**16**), **8BA4** (JAK2 JH2 V617F-**16**), **8B99** (JAK2 JH2 V617F-**1**), **8BAB** (JAK2 JH2 V617F-**4**), **8BA2** (JAK2 JH2 V617F-**23**): http://www.rcsb.org. Other data is contained within the article and the Supplementary Materials.

## Supplementary Materials

The following supporting information can be downloaded at: https://www.mdpi.com/article/10.3390/ph16010075/s1, Figure S1: Crystal structures of 23 and 4 with JAK2 JH2 V617F; Figure S2: Structural basis for compound selectivity against JAK2 JH1; Table S1: Binding of selected hits to JAK2 and JAK1 pseudokinase domains; Table S2: Chemical structures of diaminotriazole analogs and binding characteristics Binding; Table S3: Chemical structures of diaminotriazine analogs and binding characteristics; Table S4: Chemical structures of phenylpyrazolo-pyrimidone analogs and binding characteristics; Table S5: Chemical structures of pyrazolyl-formamide analogs and binding characteristics; Table S6: Effects of compounds on Ba/F3-hJAK2 wildtype and V617F cells; Table S7: Supplier details on hit compounds of this study; Table S8: Product details on hit analogs of this study; Table S9: Data collection and refinement statistics.
