# Supplementary material for: Identification of Novel Small Molecule Ligands for JAK2 Pseudokinase Domain
Source: Pharmaceuticals (Basel). 2023 Jan 4;16(1):75. doi: 10.3390/ph16010075 (PMC9865020; doi:10.3390/ph16010075)
Supplement: Supplementary file 1 [file pharmaceuticals-16-00075-s001.zip › pharmaceuticals-2104110-supplementary.pdf]

Supplementary material for:

Identification of novel small molecule ligands for JAK2 pseudokinase domain

Anniina T. Virtanen [1,2,*], Teemu Haikarainen [1], Parthasarathy Sampathkumar [3,4], Maaria Palmroth [1], Sanna Liukkonen [1], Jianping Liu [5,6], Natalia Nekhotiaeva [6,7], Stevan R. Hubbard [3] and Olli Silvennoinen [1,2,8,*]

[1] Faculty of Medicine and Health Technology, Tampere University, 33014 Tampere, Finland
[2] Institute of Biotechnology, HiLIFE Helsinki Institute of Life Science, University of Helsinki, 00014 Helsinki, Finland
[3] Department of Biochemistry and Molecular Pharmacology, New York University Grossman School of Medicine, New York, NY 10016, USA
[4] Surrozen, Inc., South San Francisco, CA 94080, USA
[5] Single Cell Core (SICOF), Department of Medicine Huddinge, Karolinska Institutet, SE-14157 Huddinge, Sweden
[6] Karolinska High Throughput Centre, Department of Biosciences and Nutrition, Karolinska Institutet, SE-14186 Huddinge, Sweden
[7] Science for Life Laboratory, Biochemical and Cellular Assay Facility, Drug Discovery and Development Platform, Department of Biochemistry and Biophysics, Stockholm University, Solna, SE-17121 Stockholm, Sweden
[8] Fimlab Laboratoriot Oy Ltd., 33013 Tampere, Finland
* Correspondence: anniina.t.virtanen@tuni.fi (A.T.V.); olli.silvennoinen@tuni.fi (O.S.)

Table of contents:

Table S1. Binding of selected hits to wild-type JAK2 pseudokinase domain. 2
Table S2. Chemical structures of diaminotriazole analogs and binding characteristics 3
Table S3. Chemical structures of diaminotriazine analogs and binding characteristics. 5
Table S4. Chemical structures of phenylpyrazolo-pyrimidone analogs and binding characteristics. 7
Table S5. Chemical structures of pyrazolyl-formamide analogs and binding characteristics. 8
Table S6. Effects of compounds on Ba/F3-hJAK2 wildtype and V617F cells 9
Table S7. Supplier details on hit compounds of this study. 10
Table S8. Product details on hit analogs of this study. 11
Table S9. Data collection and refinement statistics. 12
Figure S1. Crystal structures of 23 and 4 with JAK2 JH2 V617F. 14
Figure S2. Structural basis for compound selectivity against JAK2 JH1 15

Table S1. Binding of selected hits to wild-type JAK2 pseudokinase domain. Data presented is half-maximal inhibitory concentrations (IC50) of tracer binding to JAK2 JH2 wild-type, average ± standard deviation.

**Compound**		**JAK2 JH2 wt IC50 [µM]**
Diaminotriazole	**1**	≤ 3.5[1]
	**3**	≤ 3.5[1]
Diaminotriazine	**4**	94 ± 28
	**5**	95 ± 12
Aminopurine	**6**	≤ 3.5 [1]
	**7**	≤ 3.5 [1]
	**8**	100 ± 22
	**9**	774 ± 64
	**10**	82 ± 28
	**11**	192 ± 41
	**12**	164 ± 11
Other hits	**2**	19 ± 2
	**13**	55 ± 5
	**14**	213 ± 63
	**15**	82 ± 16

[1] at or below lower detection limit of assay.

Table S2. Chemical structures of diaminotriazole analogs and binding characteristics. General structure and side chains of the 3,5-diamino-1,2,4-triazole compounds in the study and inhibition-% of tracer binding at 10 µM and 100 µM concentrations for JAK2 JH2, JAK2 JH2 V617F, JAK2 JH1, and JAK1 JH2.

				Inhibition of tracer binding [%]							
				JAK2 JH2		JAK2 JH2 V617F		JAK2 JH1		JAK1 JH2	
**Compound**	**R1**	**R2**	**R3**	**100 µM**	**10 µM**	**100 µM**	**10 µM**	**100 µM**	**10 µM**	**100 µM**	**10 µM**
**1**, JNJ-7706621	H	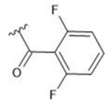	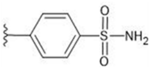	97	87	102	76	94	85	94	36
**16**, analog1	H	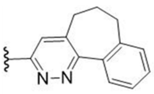	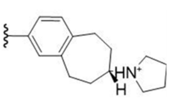	79	36	56	19	86	64	79	30
**17**, analog2	H	H	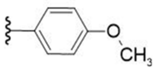	40	9	48	17	21	4	76	30
**18**, analog3	H	H	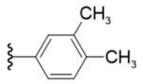	66	14	71	16	36	2	85	37
analog4	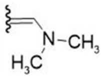	H	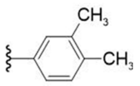	9	10	5	3	3	0	16	12
analog5	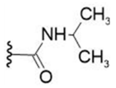	H	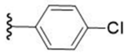	10	10	0	0	0	8	11	5
analog6	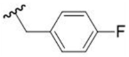	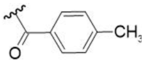	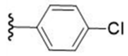	5	0	5	10	10	1	13	10
analog7	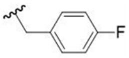	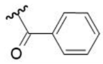	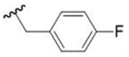	0	0	10	0	8	5	6	7
analog8	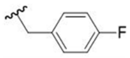	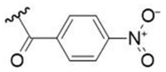	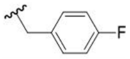	11	9	5	0	0	5	9	9
analog9	H	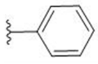	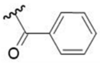	0	0	0	0	3	1	0	0
analog10	H	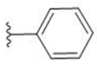	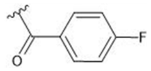	0	0	0	0	3	3	14	0
**19**, analog11	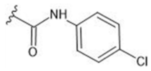	H	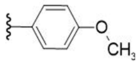	57	33	77	44	86	56	110	74

Table S2. Chemical structures of diaminotriazole analogs and binding characteristics. Continued

Compound	R1	R2	R3	Inhibition of tracer binding [%]							
				JAK2 JH2		JAK2 JH2 V617F		JAK2 JH1		JAK1 JH2	
				100 µM	10 µM	100 µM	10 µM	100 µM	10 µM	100 µM	10 µM
analog12	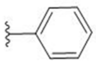	H	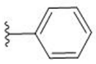	0	0	0	2	20	15	21	16
**20**, analog13	H	H	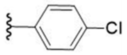	55	16	57	10	19	6	78	36
**21**, analog14	H	H	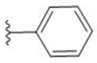	45	9	45	11	27	16	79	36
**22**, analog15	H	H	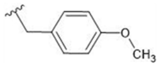	1	0	0	5	10	9	5	4

Table S3. Chemical structures of diaminotriazine analogs and binding characteristics. General structure and side chains of the 2,4-diamino-1,3,5-triazine compounds in the study and inhibition-% of tracer binding at 10 µM and 100 µM concentrations for JAK2 JH2, JAK2 JH2 V617F, JAK2 JH1, and JAK1 JH2.

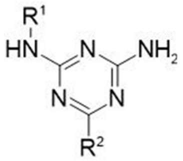

Compound	R1	R2	Inhibition of tracer binding [%]							
			JAK2 JH2		JAK2 JH2 V617F		JAK2 JH1		JAK1 JH2	
			100 µM	10 µM	100 µM	10 µM	100 µM	10 µM	100 µM	10 µM
**4**, CB_7644166	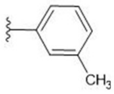	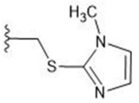	60	29	77	32	5	0	93	54
**5**, Z90271204	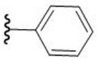	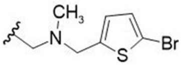	60	25	68	22	5	3	68	23
**23**, analog1	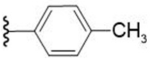	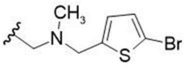	66	29	50	32	0	14	89	35
analog2	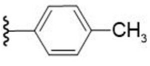	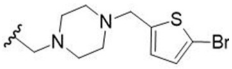	24	0	33	13	0	0	60	21
**24**, analog3	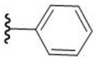	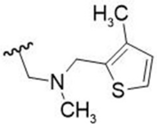	85	32	90	35	13	5	99	42
**25**, analog4	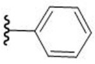	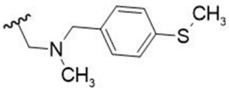	62	20	65	27	7	11	76	35
**26**, analog5	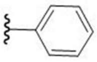	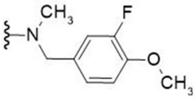	56	12	70	7	0	0	79	40
**27**, analog6	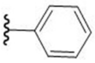	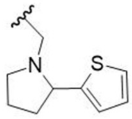	62	0	31	3	0	0	72	3
**28**, analog7	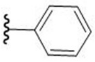	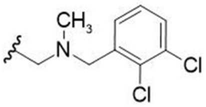	69	14	73	30	4	6	91	35
**29**, analog8	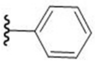	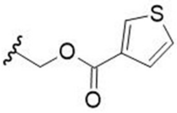	31	11	38	2	3	0	98	48
**30**, analog9	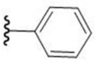	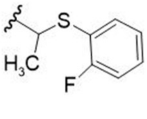	57	12	74	13	31	0	99	50
**31**, analog10	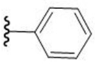	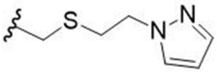	37	3	43	16	11	0	86	40

Table S3. Chemical structures of diaminotriazine analogs and binding characteristics. Continued

Compound	R1	R2	JAK2 JH2 100 µM	JAK2 JH2 10 µM	JAK2 JH2 V617F 100 µM	JAK2 JH2 V617F 10 µM	JAK2 JH1 100 µM	JAK2 JH1 10 µM	JAK1 JH2 100 µM	JAK1 JH2 10 µM
			Inhibition of tracer binding [%]							
**32**, analog11	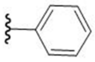	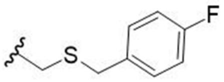	56	21	59	18	18	0	90	58
analog12	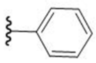	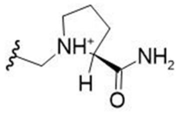	26	9	32	7	0	0	80	29
analog13	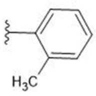	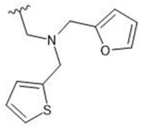	0	0	0	0	0	6	0	0
analog14	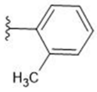	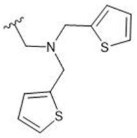	0	8	0	0	0	3	0	12
analog15	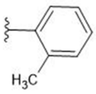	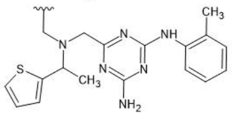	0	7	0	0	5	13	0	3
analog16	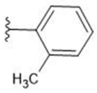	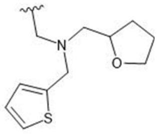	0	2	0	2	12	13	14	16
analog17	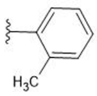	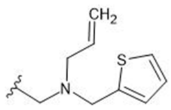	1	7	6	7	9	14	16	29
analog18	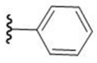	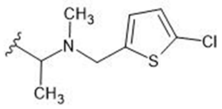	13	0	1	0	5	0	55	9
analog19	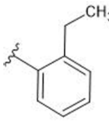	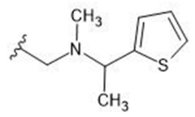	3	2	0	0	3	5	4	0
analog20	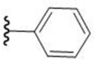	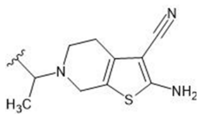	0	0	0	0	0	0	0	0
analog21	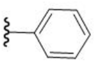	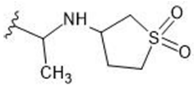	4	0	1	0	4	4	27	1
analog22	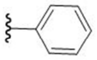	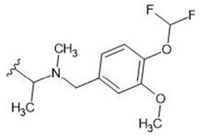	7	0	10	0	7	6	48	14
analog23	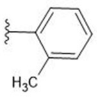	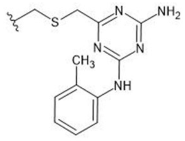	0	0	0	1	7	11	27	17
**33**, analog24	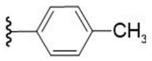	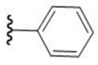	30	5	35	5	40	14	112	49

Table S4. Chemical structures of phenylpyrazolo-pyrimidone analogs and binding characteristics. General structure and side chains of the 3-phenylpyrazolo[1,5-a]pyrimidin-7(1H)-one compounds in the study and and inhibition-% of tracer binding at 10 µM and 100 µM concentrations for JAK2 JH2, JAK2 JH2 V617F, JAK2 JH1, and JAK1 JH2.

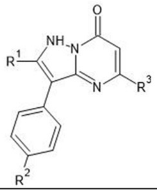

Compound	R1	R2	R3	Inhibition of tracer binding [%]							
				JAK2 JH2		JAK2 JH2 V617F		JAK2 JH1		JAK1 JH2	
				100 µM	10 µM	100 µM	10 µM	100 µM	10 µM	100 µM	10 µM
**13**, HTS01632	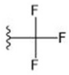	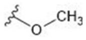	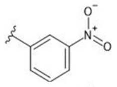	85	27	68	19	15	8	84	21
**34**, analog1	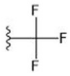	H	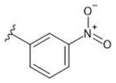	83	30	77	22	25	5	71	19
**35**, analog2	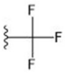	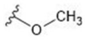	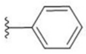	71	23	67	8	10	2	58	22
**36**, analog3	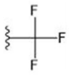	H	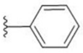	70	30	53	10	28	0	56	18
**37**, analog4	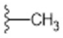	H	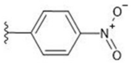	1	1	0	0	1	0	19	11
**38**, analog5	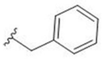	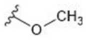	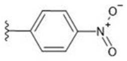	0	0	0	0	0	10	7	10
**39**, analog6	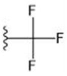	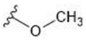	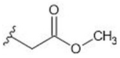	0	3	6	0	13	10	0	10
**40**, analog7	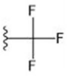	H	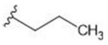	8	0	0	2	8	9	38	13
**41**, analog8	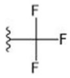	H	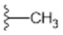	6	3	5	6	34	10	24	12
**42**, analog9	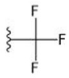	H	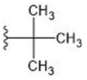	18	3	15	4	27	14	31	24

Table S5. Chemical structures of pyrazolyl-formamide analogs and binding characteristics. General structure and side chains of N-(1H-pyrazol-4-yl)formamide compounds in the study and and inhibition-% of tracer binding at 10 µM and 100 µM concentrations for JAK2 JH2, JAK2 JH2 V617F, JAK2 JH1, and JAK1 JH2.

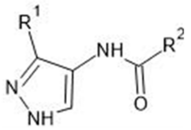

Compound	R1	R2	Inhibition of tracer binding [%]							
			JAK2 JH2		JAK2 JH2 V617F		JAK2 JH1		JAK1 JH2	
			100 µM	10 µM	100 µM	10 µM	100 µM	10 µM	100 µM	10 µM
**2**, AT-9283	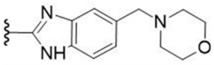	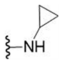	90	48	83	47	92	95	98	63
analog1	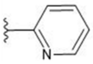	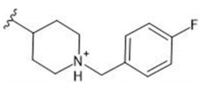	10	5	0	4	79	42	61	21
analog2	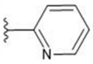	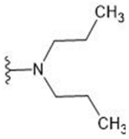	6	13	0	8	71	39	89	42
analog3	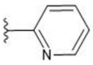	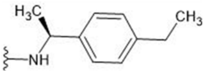	12	9	10	4	78	51	38	13
analog4	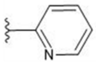	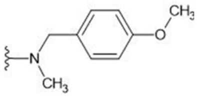	22	14	9	7	83	46	68	33
analog5	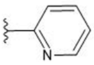	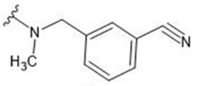	48	27	28	0	76	67	68	9
analog6	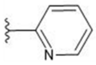	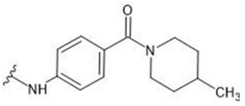	15	11	2	6	60	28	79	28
analog7	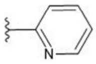	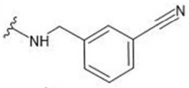	46	6	36	5	74	23	53	18
analog8	H	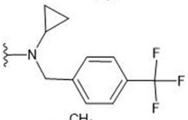	0	4	2	9	17	15	16	23
analog9	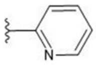	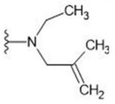	14	3	0	0	102	61	94	34

Table S6. Effects of compounds on Ba/F3-hJAK2 wildtype and V617F cells. Data is presented as IC50 [µM] of cell viability obtained by fitting dose vs. normalized response across multiple replica data sets (n=3).

		**Cell viability IC50 [µM]**	
**Compound**		**Ba/F3-hJAK2 wt**	**Ba/F3-hJAK2 V617F**
Diaminotriazole	**1**	2.6	2.7
	**18**	ND	ND
Diaminotriazine	**4**	ND	ND
	**24**	ND	ND
Aminopurine	**7**	0.38	0.48
Phenylpyrazolo-pyrimidone	**34**	ND	ND
	**36**	ND	ND
Pyrazolyl-formamide	**2**	0.33	0.67

ND, inhibition not detected or IC50>40 µM. Compound abbreviations: **1**, JNJ-7706621; **18**, JS-014C; **4**, CB_7644166; **24**, Z53013220; **7**, Reversine; **34**, HTS02998; **36**, HTS02993; **2**, AT-9283.

Table S7. Supplier details on hit compounds of this study.

Compound	***Supplier***
JNJ-7706621	Selleck Chemicals
Cdk1/2 inhibitor III	Merck Millipore
CB_7644166	ChemBridge Corporation
Z90271204	Enamine Ltd
Cdk2 inhibitor IV (NU6140)	Merck Millipore
Reversine	Cayman Chemical
F0578-0019	Life Chemicals
F0578-0023	Life Chemicals
F0578-0149	Life Chemicals
F0578-0155	Life Chemicals
F0578-0135	Life Chemicals
AT9283	Selleck Chemicals
HTS01632	Maybridge, Ltd.
Gandotinib (LY2784544)	Selleck Chemicals
AZD7762	Selleck Chemicals

Table S8. Product details on hit analogs of this study.

Analog	Product ID	Supplier
Diaminotriazole analog1	R428	Selleck Chemicals
Diaminotriazole analog2	TG00127	Maybridge, Ltd.
Diaminotriazole analog3	JS-014C	Key Organics Ltd
Diaminotriazole analog4	JS-1368	Key Organics Ltd
Diaminotriazole analog5	TG00133	Maybridge, Ltd.
Diaminotriazole analog6	STK187588	Vitas M Chemical Ltd
Diaminotriazole analog7	STK178876	Vitas M Chemical Ltd
Diaminotriazole analog8	STK187595	Vitas M Chemical Ltd
Diaminotriazole analog9	STK846475	Vitas M Chemical Ltd
Diaminotriazole analog10	STK060079	Vitas M Chemical Ltd
Diaminotriazole analog11	TG00130	Maybridge, Ltd.
Diaminotriazole analog12	AE-484/30082023	Specs
Diaminotriazole analog13	JS-158C	Key Organics Ltd
Diaminotriazole analog14	TG00124	Maybridge, Ltd.
Diaminotriazole analog15	F235-0452	ChemDiv, Inc.
Diaminotriazine analog1	Z53013220	Enamine Ltd
Diaminotriazine analog2	Z52738116	Enamine Ltd
Diaminotriazine analog3	Z90249435	Enamine Ltd
Diaminotriazine analog4	Z90228515	Enamine Ltd
Diaminotriazine analog5	Z90237919	Enamine Ltd
Diaminotriazine analog6	Z90226552	Enamine Ltd
Diaminotriazine analog7	Z90223815	Enamine Ltd
Diaminotriazine analog8	Z90110078	Enamine Ltd
Diaminotriazine analog9	Z193554240	Enamine Ltd
Diaminotriazine analog10	Z1403623362	Enamine Ltd
Diaminotriazine analog11	Z99558383	Enamine Ltd
Diaminotriazine analog12	Z90204970	Enamine Ltd
Diaminotriazine analog13	Z89071621	Enamine Ltd
Diaminotriazine analog14	Z106434712	Enamine Ltd
Diaminotriazine analog15	PB203130188	UkrOrgSynthesis Ltd
Diaminotriazine analog16	Z90267928	Enamine Ltd
Diaminotriazine analog17	Z89205406	Enamine Ltd
Diaminotriazine analog18	Z195726712	Enamine Ltd
Diaminotriazine analog19	Z195938424	Enamine Ltd
Diaminotriazine analog20	Z1540446168	Enamine Ltd
Diaminotriazine analog21	Z198049298	Enamine Ltd
Diaminotriazine analog22	Z195616332	Enamine Ltd
Diaminotriazine analog23	Z45354386	Enamine Ltd
Diaminotriazine analog24	35080465	ChemBridge Corporation
Phenylpyrazolo-pyrimidone analog1	HTS02998	Maybridge, Ltd.
Phenylpyrazolo-pyrimidone analog2	HTS01428	Maybridge, Ltd.
Phenylpyrazolo-pyrimidone analog3	HTS02993	Maybridge, Ltd.
Phenylpyrazolo-pyrimidone analog4	5669620	ChemBridge Corporation
Phenylpyrazolo-pyrimidone analog5	5670982	ChemBridge Corporation
Phenylpyrazolo-pyrimidone analog6	HTS01631	Maybridge, Ltd.
Phenylpyrazolo-pyrimidone analog7	HTS02995	Maybridge, Ltd.
Phenylpyrazolo-pyrimidone analog8	STK850741	Vitas M Chemical Ltd
Phenylpyrazolo-pyrimidone analog9	HTS02994	Maybridge, Ltd.
Pyrazolyl-formamide analog1	Z1453153362	Enamine Ltd
Pyrazolyl-formamide analog2	Z1567473124	Enamine Ltd
Pyrazolyl-formamide analog3	Z1453154041	Enamine Ltd
Pyrazolyl-formamide analog4	Z1539628219	Enamine Ltd
Pyrazolyl-formamide analog5	Z1539628308	Enamine Ltd
Pyrazolyl-formamide analog6	Z1567475331	Enamine Ltd
Pyrazolyl-formamide analog7	Z1539628112	Enamine Ltd
Pyrazolyl-formamide analog8	Z815279816	Enamine Ltd
Pyrazolyl-formamide analog9	Z369935268	Enamine Ltd

Table S9. Data collection and refinement statistics.

	WT-7 (PDB code 8EX1)	V617F-7 (PDB code 8BAK)	WT-6 (PDB code 8EX0)	V617F-6 (PDB code 8B8N)
**Data**				
Beam line	APS 19-BM	Diamond I03	APS 19-BM	Diamond I03
Wavelength (Å)	0.9792	0.97625	0.9794	0.97625
Space group	C2	$P2_12_12_1$	$P2_1$	$P2_12_12_1$
Cell dimensions				
a, b, c (Å)	91.08, 57.71, 60.75	53.28, 56.96, 114.36	43.98, 57.57, 61.18	57.07, 61.16, 91.13
α, β, γ (°)	90.00, 94.32, 90.00	90.00, 90.00, 90.00	90.00, 110.38, 90.00	90.00, 90.00, 90.00
Resolution (Å)	50-1.50 (1.53-1.50)	57-1.65 (1.75-1.65)	29-1.85 (1.88-1.85)	45-2.00 (2.12-2.00)
$CC_{1/2}$ (%)	99.9 (89.9)	99.9 (48.4)	99.8 (84.1)	99.9 (76.4)
$I/\sigma I$	17.1 (4.0)	16.03 (1.20)	13.9 (3.2)	10.11 (1.32)
R_{merge}	0.055 (0.433)	0.067 (1.569)	0.062 (0.598)	0.110 (1.582)
Completeness (%)	99.7 (99.1)	100 (99.8)	99.9 (100)	100 (99.9)
Redundancy	4.9 (4.6)	7.1 (7.1)	3.8 (3.7)	7.1 (7.3)
**Refinement**				
Reflections	50203	80988	24548	41624
R_{work}/R_{free}	0.169/0.191	0.184/0.207	0.175/0.228	0.209/0.255
RMSD of bond lengths (Å)	0.010	0.012	0.011	0.004
RMSD of bond angles (°)	1.02	0.93	1.05	0.54
B-factors (Å^2)				
Protein	22.9	39.0	37.3	68.8
Ligand	16.6	60.5	36.5	75.6

Values for the highest-resolution shell are shown in parentheses.

Table S9. Data collection and refinement statistics. Continued

	WT-36 (PDB code 8EX2)	V617F-36 (PDB code 8B8U)	WT-24 (PDB code 8B9H)	V617F-24 (PDB code 8B9E)
**Data**				
Beam line	APS 19-BM	Diamond I03	Diamond I03	Diamond I03
Wavelength (Å)	0.9792	0.97625	0.97625	0.97625
Space group	C2	P1	$P2_1$	$P2_12_12_1$
Cell dimensions				
a, b, c (Å)	92.16, 57.42, 60.52	46.27, 56.80, 60.65	44.18, 57.08, 61.37	53.46, 56.55, 114.93
α, β, γ (°)	90.00, 94.55, 90.00	89.97, 79.14, 71.39	90.00, 110.95, 90.00	90.00, 90.00, 90.00
Resolution (Å)	27-1.90 (1.93-1.90)	41-1.50 (1.53-1.50)	57-1.50 (1.59-1.50)	57-1.50 (1.59-1.50)
$CC_{1/2}$ (%)	99.6 (89.1)	99.3 (80.0)	99.9 (68.0)	99.9 (91.5)
$I/\sigma I$	14.8 (4.1)	9.20 (1.10)	12.23 (1.05)	13.68 (0.77)
R_{merge}	0.087 (0.629)	0.061 (0.849)	0.044 (1.006)	0.068 (1.310)
Completeness (%)	99.8 (99.8)	94.9 (76.2)	95.9 (88.7)	91.6 (61.5)
Redundancy	4.5 (4.4)	3.5 (3.1)	3.6 (3.3)	6.1 (3.4)
**Refinement**				
Reflections	24886	86906	86138	98868
R_{work}/R_{free}	0.169/0.203	0.222/0.259	0.181/0.209	
RMSD of bond lengths (Å)	0.012	0.007	0.009	0.017
RMSD of bond angles (°)	1.11	0.89	0.89	1.27
B-factors				
Protein	26.6	29.7	37.4	30.5
Ligand	18.9	21.0	32.6	23.0

Values for the highest-resolution shell are shown in parentheses.

Table S9. Data collection and refinement statistics. Continued

	WT-16 (PDB code 8BA3)	V617F-16 (PDB code 8BA4)	V671F- 1 (PDB code 8B99)	V617F-4 (PDB code 8BAB)
**Data**				
Beam line	Diamond I03	Diamond I03	Diamond I03	Diamond I03
Wavelength (Å)	0.97625	0.97625	0.97625	0.97625
Space group	C2	P1	$P2_12_12_1$	$P2_12_12_1$
Cell dimensions				
a, b, c (Å)	78.22, 57.75, 60.63	55.57, 57.09, 60.70	53.41, 56.72, 114.50	53.24, 56.80, 114.75
α, β, γ (°)	90.00, 91.62, 90.00	90.06, 116.01, 115.65	90.00, 90.00, 90.00	90.00, 90.00, 90.00
Resolution (Å)	60-1.4 (1.49-1.40)	53-2.10 (2.23-2.10)	40-1.60 (1.70-1.60)	57-1.55 (1.64-1.55)
$CC_{1/2}$ (%)	99.9 (43.9)	99.5 (46.1)	100 (36.5)	99.9 (63.8)
$I/\sigma I$	12.48 (1.00)	6.62 (1.13)	16.41 (0.83)	11.93 (1.42)
R_{merge}	0.048 (0.761)	0.08 (0.701)	0.056 (1.486)	0.095 (1.202)
Completeness (%)	95.1 (72.6)	95.3 (95.2)	96.6 (81.6)	99.9 (99.2)
Redundancy	3.3 (2.1)	1.8 (1.9)	6.6 (4.7)	7.1 (6.9)
**Refinement**				
Reflections	99229	65710	85667	97528
R_{work}/R_{free}	0.174/0.197	0.216/0.274	0.189/0.211	0.175/0.203
RMSD of bond lengths (Å)	0.016	0.003	0.006	0.019
RMSD of bond angles (°)	1.27	0.60	0.88	1.37
B-factors				
Protein	25.5	49.1	34.9	29.2
Ligand	30.2	66.8	32.6	24.8

Values for the highest-resolution shell are shown in parentheses.

Table S9. Data collection and refinement statistics. Continued

	V617F-23 (PDB code 8BA2)
**Data**	
Beam line	Diamond I03
Wavelength (Å)	0.97625
Space group	$P2_12_12_1$
Cell dimensions	
a, b, c (Å)	53.24, 56.76, 114.74
α, β, γ (°)	90.00, 90.00, 90.00
Resolution (Å)	57-1.50 (1.59-1.50)
$CC_{1/2}$ (%)	99.9 (61.9)
$I/\sigma I$	13.64 (1.46)
R_{merge}	0.075 (1.061)
Completeness (%)	100 (99.8)
Redundancy	6.9 (5.9)
**Refinement**	
Reflections	107715
R_{work}/R_{free}	0.177/0.204
RMSD of bond lengths (Å)	0.016
RMSD of bond angles (°)	1.25
B-factors	
Protein	26.3
Ligand	20.1

Values for the highest-resolution shell are shown in parentheses.

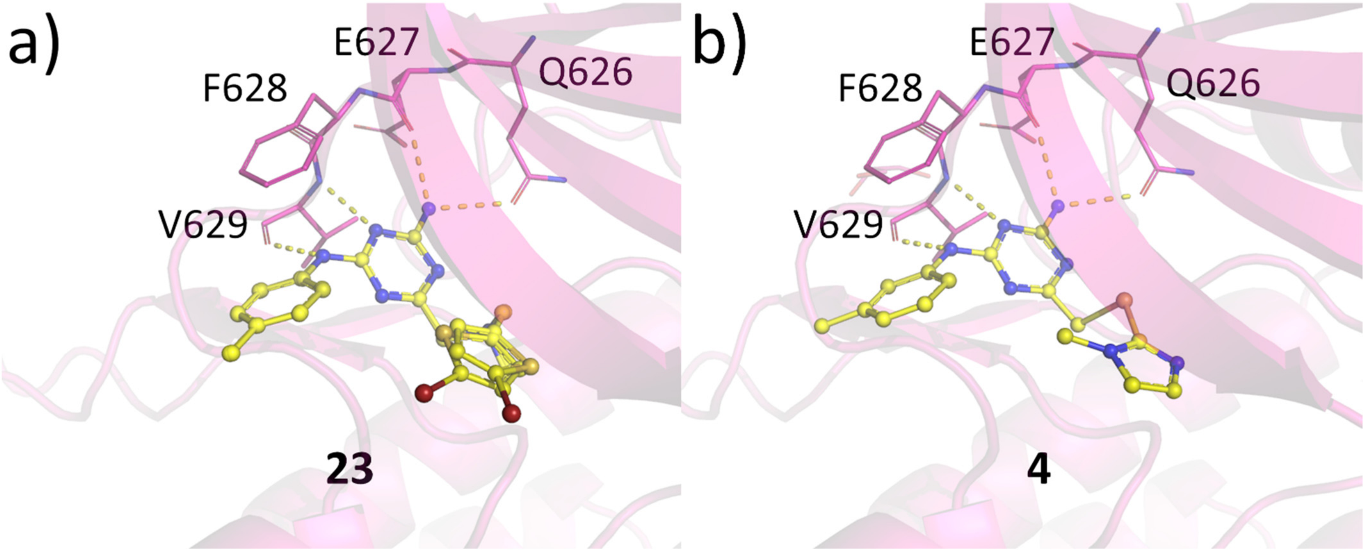

Figure S1. Crystal structures of 23 and 4 with JAK2 JH2 V617F. The residues participating in hydrogen bonding are shown as sticks. The hydrogen bonds are shown in yellow dotted lines. a) **23**. b) **4**.

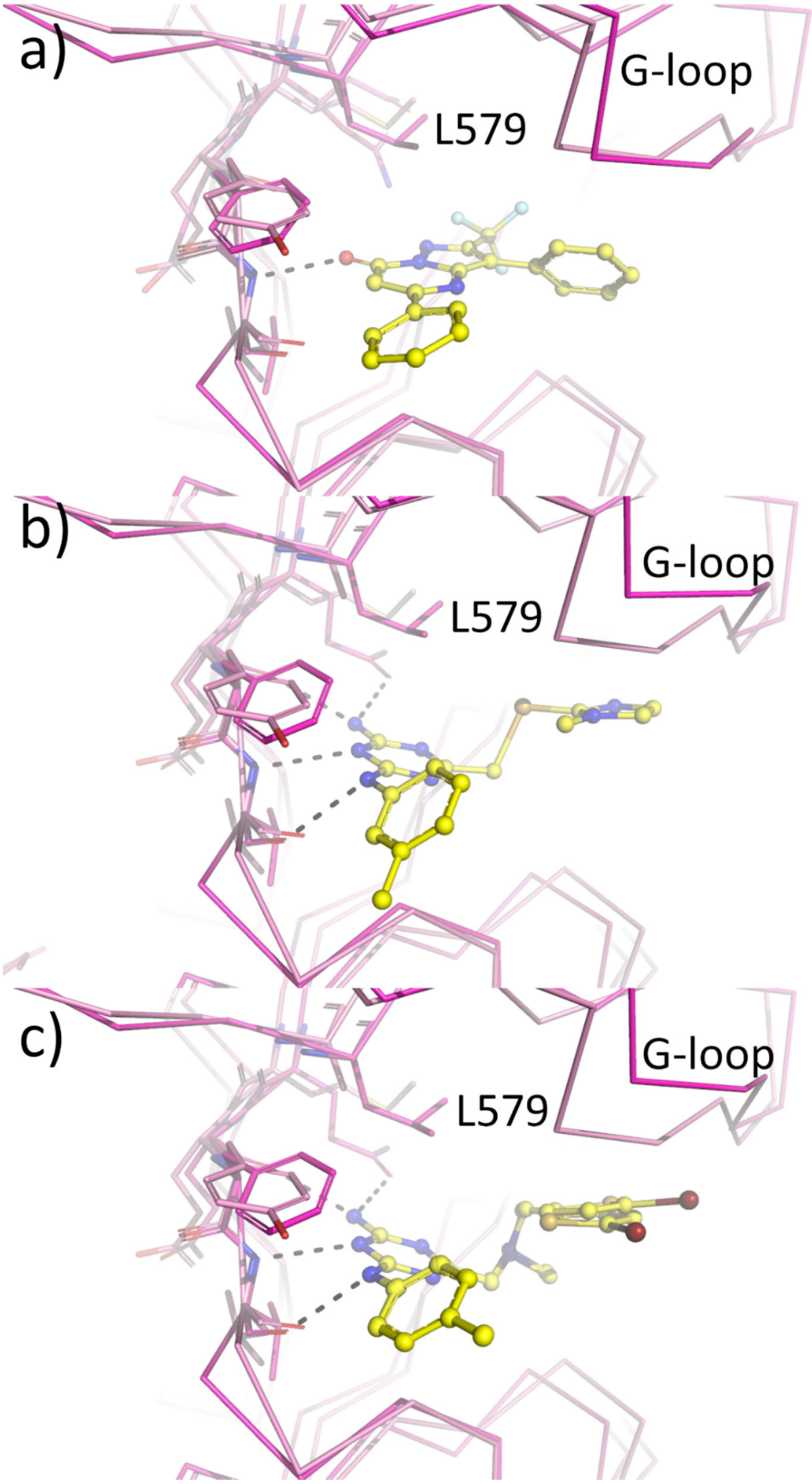

Figure S2. Structural basis for compound selectivity against JAK2 JH1 (pdb coe 4IVA). a) JAK2 JH2 V617F-**37** superposed with JAK2 JH1. b) JAK2 JH2 V617F-**4** superposed with JAK2 JH1. c) JAK2 JH2 V617F-**23** superposed with JAK2 JH1. JAK2 is colored in magenta and JAK1 is colored in pink. Hydrogen bonds are depicted as dotted lines.
